# Implementation of a digital behavior change intervention (eCHANGE) for weight loss maintenance support: a service design and technology transfer approach

**DOI:** 10.3389/fdgth.2024.1394599

**Published:** 2024-07-02

**Authors:** R. A. Asbjørnsen, J. Hjelmesæth, M. L. Smedsrød, J. Wentzel, M. M. Clark, S. M. Kelders, J. E. W. C. van Gemert-Pijnen, L. Solberg Nes

**Affiliations:** ^1^ Centre for eHealth and Wellbeing Research, Section of Psychology, Health & Technology, Department of Technology, Human and Institutional Behaviour, Faculty of Behavioural, Management and Social Sciences, University of Twente, Enschede, Netherlands; ^2^ Research and Innovation Department, Vestfold Hospital Trust, Tønsberg, Norway; ^3^ Department of Digital Health Research, Division of Medicine, Oslo University Hospital, Oslo, Norway; ^4^ Department of Endocrinology, Obesity and Nutrition, Vestfold Hospital Trust, Tønsberg, Norway; ^5^ Institute of Clinical Medicine, Faculty of Medicine, University of Oslo, Oslo, Norway; ^6^ Collaborative Care Unit, Sørlandet Hospital Trust, Kristiansand, Norway; ^7^ Research Group IT Innovations in Health Care, Windesheim University of Applied Sciences, Zwolle, Netherlands; ^8^ Department of Psychiatry & Psychology, College of Medicine & Science, Mayo Clinic, Rochester, MN, United States

**Keywords:** eHealth, digital behavior change intervention, implementation, technology transfer, service design, innovation, obesity, weight loss maintenance

## Abstract

Obesity is a chronic disease, and while weight loss is achievable, long-term weight loss maintenance is difficult and relapse common for people living with obesity. Aiming to meet the need for innovative approaches, digital behavior change interventions show promise in supporting health behavior change to maintain weight after initial weight loss. Implementation of such interventions should however be part of the design and development processes from project initiation to facilitate uptake and impact. Based on the development and implementation process of eCHANGE, an evidence-informed application-based self-management intervention for weight loss maintenance, this manuscript provides suggestions and guidance into; (1) How a *service design approach* can be used from initiation to implementation of digital interventions, and (2) How a *technology transfer process* can accelerate implementation of research-based innovation from idea to market.

## Introduction

Health systems worldwide are facing challenges related to the health and economic burden of chronic diseases such as obesity (1–3), and ongoing innovation and introduction of new technologies offering low-cost, wide-reaching approaches are required to meet the needs of patients and healthcare providers (4–7). Behavioral lifestyle interventions, single-standing or in conjunction with other treatment modalities (e.g., anti-obesity medication, surgery), are recommended by clinical practice guidelines for obesity management in order to lose weight and prevent weight regain (8–11). However, few people living with obesity are successful in maintaining reduced weight to attain health benefits over time (12, 13). Long-term weight loss maintenance is hence arising as a major healthcare goal, and innovative solutions are needed (5, 14, 15).

Digital behavior change (i.e., eHealth) interventions are promising solutions for supporting self-management and healthy lifestyle changes to maintain weight and improve health (16–19).^1^ The advantage of a digital [e.g., application (app)-based] behavior change program for weight loss maintenance, alone, or in combination with other treatment forms, would be the adaptable and scalable capabilities to support individual needs across time and context (20, 21). As such, the potential of digital health interventions and blended care models (i.e., combination of technology and face-to-face services) (22) has been recognized by The World Health Organization's (WHO) policy considerations and guideline recommendations for prevention and management of obesity (5, 9).

### Implementation and technology transfer of digital interventions

Introduction of new digital interventions can change healthcare processes (e.g., patient pathways) and contribute to improve efficiency, quality of care and health outcomes. Existing health services can also influence how new digital health solutions are designed, and implementation of digital interventions can influence how healthcare is organized and delivered (22, 23). For digital health interventions to have an impact on care and outcomes, however, they must be adopted and integrated into the healthcare system (22). Successful implementation of new technologies in healthcare can be complex though, and a number of approaches have been suggested (22, 24, 25). Technology transfer (i.e., transfer of knowledge from research into products and services) and adoption of evidence-informed inventions can be a time-consuming and slow process, and there is often a gap between the actual invention, implementation and diffusion (i.e., natural spread) (26–31). Implementation, often seen as a post-design activity, should in fact be incorporated in the design and development process of new technologies to ensure a fit with patient and healthcare provider needs and preferences, as well as the healthcare context (32, 33). To address the gap between science and translation of research-based innovations into clinical practice, feasible methods to enhance implementation and delivery of evidence-informed solutions for obesity management are required (34).

Implementation of digital health interventions can be defined as the activities undertaken to achieve adoption and use of a technology in the intended context (22). Even though various frameworks and theoretical concepts exist to guide such implementation (22, 32), few studies have yet described practical approaches that can be successfully applied to address implementation and wide-spread use of evidence-informed digital interventions in healthcare (35–37). Research and development of new digital health technologies should therefore provide in-depth insights into the process from idea to implementation to facilitate knowledge transfer and increase adoption and effectiveness of digital interventions. This manuscript seeks to provide suggestions and strategies for how *service design* and *technology transfer* of research-based innovation can facilitate implementation of an app-based digital behavior change intervention for weight loss maintenance into healthcare.

### Approaches to implementation

Service design is one approach that can be used to address the issue of implementation during the design and development process of new technologies. Service design is a human-centered, multidisciplinary, and holistic approach that can be used to achieve insight into existing health services, needs and experiences of stakeholders in order to improve, redesign or create new services and solutions (38). As such, service design can aid in matching the context, needs, and technology to facilitate implementation of new technology-enabled services (38–40).

Technology transfer processes, where innovations are translated into solutions and made available for the public through commercialization (41), might also facilitate the implementation of new digital interventions into healthcare. A Technology Transfer Office can support the process of bringing new research-based ideas to the market (41, 42) by evaluating the business potential of an invention, including regulatory compliance and the possibilities to actually define a viable product based on a Disclosure of Invention (DOFI). More knowledge related to how technology transfer processes may facilitate implementation of digital health interventions into healthcare practice is however needed.

### Study details

To address the need for innovative approaches to successful weight loss maintenance, an evidence-informed app-based self-management behavior change intervention called eCHANGE was created to support people living with obesity in maintaining their weight after weight loss (20, 21, 43, 44). In the forthcoming sections, experiences and perspectives from research related to the eCHANGE intervention are provided regarding: (1) how a *service design approach* can be used from initiation to implementation of digital interventions, and (2) how a *technology transfer process* can accelerate implementation of research-based innovation from idea to market.

An interdisciplinary research- and development team at the Department of Digital Health Research (DIG), Oslo University Hospital (OUH) was, in collaboration with representatives from two other public hospitals in Norway (i.e., Vestfold Hospital Trust (VHT) and Sørlandet Hospital Trust (SHT)), responsible for the development, implementation and evaluation of eCHANGE (21). The process was guided by the Double Diamond (i.e., design thinking process) (45) and the Center for eHealth Research and Disease Management (CeHRes) Roadmap (22, 33), seeking to develop and deliver a desirable, feasible, and viable digital behavior change intervention (20, 21, 46–48). The systematic and iterative design and development process of eCHANGE, included evidence-based theories and strategies, innovative approaches and continuous formative evaluation cycles, to optimize and align the technology with end user values and needs (20, 21). A scoping review first identified behavior change techniques (BCTs) and persuasive system design (PSD) principles in existing eHealth interventions, to support motivation and adherence for long-term weight loss maintenance (44). Individual interviews, focus groups and workshops with end users and other stakeholders were then conducted as part of a contextual inquiry and value specification phase (20). This was done to identify which key stakeholders to involve, values and needs of end users seeking to maintain weight, and matching BCTs, PSD principles and design requirements for a digital weight loss maintenance intervention (20). In a design and operationalization phase, BCTs and PSD principles were then combined and translated into various low- and high-fidelity prototypes through ideation and co-design activities with end users and other key stakeholders (21). The prototypes were then iteratively tested and evaluated by end users and other key stakeholders to select intervention components and design features, eventually resulting in the development of eCHANGE (21). As part of the summative evaluation of eCHANGE, a feasibility pilot study was conducted (i.e., in accordance with the United Kingdom Medical Research Council's guidance framework for developing and evaluating complex interventions) (49) with participants from three secondary or tertiary obesity research and treatment centers (i.e., public hospitals) to evaluate user experience, system use and preliminary efficacy.^1^ The results from this mixed methods feasibility study are promising with respect to usability and usefulness, and to facilitate self-regulation and maintenance of health behaviors to prevent weight regain.^1^

In preparation for effective implementation and scale-up of eCHANGE, the approaches presented in this manuscript were initiated early in the design and development process, during the discover and define phase, including the contextual inquiry and value specification of the Double Diamond and CeHRes Roadmap (20, 33, 45).

### A service design approach—from initiation to implementation of digital interventions

Service design methods (38, 39) were applied during the design and development of eCHANGE to gain insight into the existing health services and optimize the fit between the needs, context, and technology to be developed (21). A service design workshop (39, 50) was performed with key stakeholders (e.g., user representatives, healthcare providers, eHealth researchers, and content editors) (20, 21). The aim of the workshop was twofold; (a) to map the current patient journey for people living with obesity (i.e., adults, BMI ≥ 30 kg/m^2^) aiming to lose weight and keep the weight off, and (b) to explore how a digital intervention could be incorporated into the health services.

During the workshop, stakeholders collaborated to create a visual overview of the current health services for people living with obesity when referred by a general practitioner to secondary or tertiary obesity research and treatment centers in Norway (21). Ideas to develop and implement a digital behavior change intervention to prevent weight regain after weight loss were also explored (21), as were ideas for service innovation and new (i.e., digital) healthcare delivery models (e.g., virtual coach and digital hub across service levels and sectors).

The service design workshop resulted in creation of a service blueprint (40, 50), visualizing the existing health services from a user perspective (See Supplementary Material S1). The service blueprint included the patient journey, needs and experiences, the perspectives of healthcare providers, touch-points, and challenges. As indicated in Supplementary Material S1, the patient journey for people living with obesity often involves multiple interactions with a range of healthcare providers across health service levels and sectors (e.g., general practitioner, medical specialist, nurse, dietitian, physiotherapist, and psychologist). Workshop participants highlighted that people living with obesity often feel left alone and unsupported after successful weight loss, with few health services and treatments available to provide professional guidance and support patient needs for maintaining weight after weight loss (21).

The service blueprint created served as basis to explore the added value of the technology (e.g., self-management support, 24/7 availability, maintenance of health behaviors) and how the digital intervention could be integrated and implemented into the healthcare system (20, 21). The stakeholder information helped to acquire a context overview, and to optimize the match between the technology, identified needs, and existing healthcare processes. To create a desirable and feasible evidence-informed digital intervention, the ensuing iterative process included multiple stakeholder involved formative evaluation cycles, as presented in the Study Detail section above. A combination of behavioral science, human-centered and agile approaches to innovation and design for behavior change was applied during the design and development process of eCHANGE (20, 21). In preparation for implementation of eCHANGE into healthcare, the feasibility pilot study^1^ was subsequently also conducted to ensure the needs of the target group, the technology and the real-world context were aligned and considered beneficial.

### A technology transfer process—from idea to market

**Figure 1:**
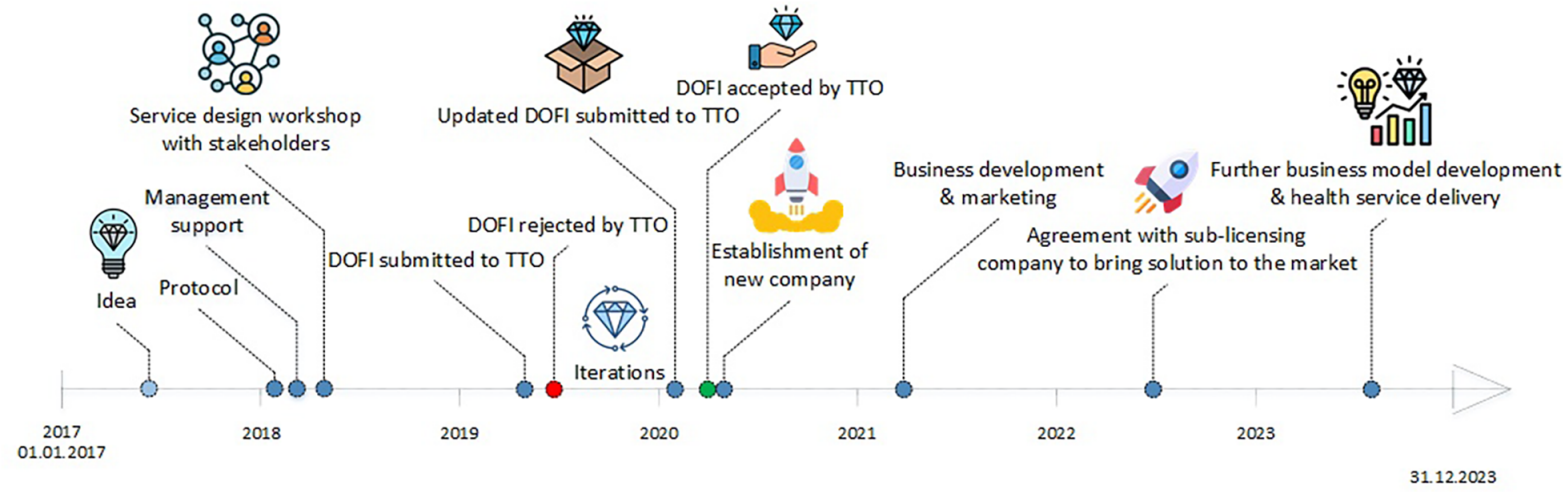
A research-based innovation approach: the technology transfer process of eCHANGE.

A Technology Transfer Office (i.e., TTO) assisted with the technology transfer process of eCHANGE. To comply with guidelines and regulations for commercialization within the Norwegian public health system (51), a DOFI was submitted to the TTO by the Principal Investigator (PI) at DIG, OUH, in collaboration with VHT and SHT, early in the design and development process. The aim of the technology transfer process, was to explore whether the idea of a digital behavior change intervention for weight loss maintenance could be commercially viable for technology transfer to the market (See Figure 1 and Supplementary Material S2).

A TTO-team consisting of experts within business development, patenting, and law assessed the commercial potential and compliance with national and international standards and regulations based on the DOFI submitted (21). The assessment also included analysis of market needs, advantages compared to competing products, patenting or protective mechanisms, and development risks. As indicated in Figure 1, the initial DOFI was rejected by the TTO as the intervention program was deemed not sufficiently developed to be assessed. After feedback from the TTO and further design and development iterations, a second DOFI was submitted with further value specification (i.e., at technology readiness level #4) (52), resulting in a positive TTO evaluation. This initiated a market search for investors interested in establishing a new company to commercialize health apps developed by DIG. An investor was selected based on the markets- and finance strategy presented, resulting in the establishment of a new company (i.e., TTO ownership; 51%). The TTO contributed with legal advice, business model development, and establishment of a board of directors and management team, as well as license transfer to the new company regulating the commercial rights to promote and provide access to eCHANGE. The process from the acceptance of the DOFI until the license agreement was established, took about 1 year.

Before bringing eCHANGE to market, the inventors and the TTO had to ensure patient safety and technology compliance with relevant laws and regulatory requirements, including medical device regulations for software (53). eCHANGE is not considered a medical device and is currently promoted as a self-management and health support program (i.e., currently at technology readiness level #8) (52) that complies with national and international standards and regulations for privacy and security, including national IT infrastructure (21). Technical aspects related to development of eCHANGE to prepare for implementation, such as risk assessment, and compliance with national and international standards and regulations (e.g., privacy, security, ethical, medical device) have been described in publications elsewhere (20, 21).^1^

The newly established company subsequently entered into a collaboration with another Norwegian company focusing on treatment of lifestyle related diseases. As indicated in Figure 1, these companies (i.e., the commercialization company and the sub-licensing company) are in the process of bringing eCHANGE to the market through a license type agreement. eCHANGE will then be incorporated into a patient-provider healthcare portal made available through healthcare community establishments (i.e., public-private partnerships), allowing for patient-provider communication, data sharing and mutual program progress viewing. DIG continues to technically maintain eCHANGE, but as the implementation process progresses and moves beyond research focus, data management, program optimization, improvement and maintenance will be transferred to the newly established companies (i.e., depending on final business model). Additional business models are also being investigated, particularly related to integration of the program into the Norwegian national, public health platform (54) and the Norwegian health finance system (55), as this could facilitate access to eCHANGE for all public healthcare providers (i.e., primary and specialist care).

## Discussion and lessons learned

The manuscript demonstrates how *service design* can be used to explore and optimize the fit and value when implementing new technologies into an existing healthcare system, and how a *technology transfer approach* can facilitate commercialization and implementation.

The overall aim of the eCHANGE project was to add knowledge to future weight loss maintenance practice through the use of digital technology, fostering service- and research-driven innovation across sectors and service levels. The *service design methods* and *technology transfer process* applied might help bridge the gap between research and implementation of innovative solutions in healthcare.

In the example of eCHANGE, the service design methods allowed for early stakeholder involvement to explore how a digital solution could be integrated and implemented into health services, as well as ideas for service innovation and new (i.e., digital) healthcare delivery models. The early involvement of stakeholders and interdisciplinary collaboration during the development of eCHANGE, including experts from a TTO, accelerated the innovation process of bringing a new idea to market. The acceptance of the DOFI by the TTO allowed the eCHANGE research and development team to mainly focus on research activities to create a desirable and feasible solution (i.e., problem-solution-fit) (21), while the TTO and the new company, with input from the inventors, could focus on viability (i.e., product-market fit) through business plan development, payment models and marketing plans. In contrast, this process might also create friction, as the technology transfer process often is a separate activity managed by TTOs. Research-inventors might experience “loss of control” and potential trade-off must be addressed upfront (i.e., before submitting a DOFI). When a DOFI is accepted by a TTO, public health inventors often have to “let go” of the intervention, as the plan is to make the technology accessible through the market, often without the involvement or say of the inventors, unless they themselves are part of the “implementation/commercialization into market” process (e.g., as stock holders or new company employees). TTO involvement during the early development can also assist research-inventors in overcoming challenges known as the “valley of death” (e.g., technology readiness, lack of financial resources and expertise between discovery and commercialization), and to bridge the innovation gap from idea to market (56).

Demonstrating the potential impact and uptake of new technology can be time- and resource-consuming (26, 57), and might influence the competitive advantage of an innovative idea. For successful research-based entrepreneurship through technology transfer, alignment of the capabilities and interests of key stakeholders (58), and collaboration between two or more institutions (e.g., academic institutions, industry, and government) in early design phase, can be important (59). Efforts to implement innovative solutions that promote sustainable behavior change to prevent and manage obesity in society might, according to WHO, therefore require public-private collaborations between researchers, healthcare providers, policy-makers, technology companies, patient organizations, and other stakeholders (5). Interdisciplinary collaboration between researchers, healthcare organizations, commercial companies, and other key stakeholders, might also contribute to integration of multiple methods and development of new approaches to facilitate implementation of innovative solutions in healthcare.

Implementation also depends on, and might be impacted by, rapid changes required or occurring. The Covid-19 pandemic is an example of a changing healthcare landscape, where digital health technologies were implemented at high speed to help patients and healthcare workers worldwide (60–62). This indicates that various factors might influence implementation of innovations and healthcare transformation, such as laws, reimbursements and infrastructure to support new ways of working (25).

Successful implementation of new digital health technologies requires, therefore, a holistic and multidisciplinary approach, addressing factors such as healthcare processes, regulations, and forms of reimbursement (24, 32, 33, 63). The service design and technology transfer approach presented can help to address these factors, foster collaboration between designers, researchers and businesses, and facilitate innovation of existing service systems (64, 65). Given the rapid technology developments and urgency of implementing innovative solutions in healthcare, iterative approaches, interdisciplinary stakeholder collaboration, and multi-method assessment, as in the eCHANGE project (21),^1^ can also contribute to minimizing the risks and costs of implementing an innovation into practice (23).

The frameworks applied and approaches presented are often used separately by designers and researchers worldwide (22, 38, 41, 57, 66). The combined approach used in this project is however, to the best of our knowledge, novel. As implementation of digital health interventions is complex, “one size does not fit all” and application of a range of frameworks and participatory methods, facilitating a holistic view of the relationship between stakeholders, technology and context, might be recommended (21, 22, 32, 33). Technology should also allow for adaptations during implementation to fit a certain setting and context (i.e., for a “product-market fit”) (21, 22, 25, 67).

### Strengths and limitations

This manuscript centers around the activities undertaken to facilitate implementation of eCHANGE, an evidence-informed app-based self-management program for weight loss maintenance (20, 21),^1^ within a public health care system. As context always matters, the experiences from eCHANGE only serves an example. Also, evaluation of the implementation with respect to uptake and impact of eCHANGE into practice was not the aim of the current manuscript (20, 21).^1^

Digital behavior change interventions, such as eCHANGE, might be cost-effective, engaging and accessible solutions for personalized behavior change support to improve weight and health outcomes of people living with long-term conditions, such as obesity (6, 17, 21). The eCHANGE app can therefore help fulfill a societal need. Even though implementation can still be challenging, the service design and technology transfer approaches applied in the eCHANGE project helped identify existing needs and an implementation strategy. Early involvement of TTOs might also help bridge the gap between development and implementation of research-based innovations into practice, but should not control the thorough development, implementation and evaluation processes required to implement effective interventions in healthcare.

### Recommendations and future directions

Further research is required to determine whether the presented approaches serve as a best practice example to facilitate implementation of innovation in healthcare. To facilitate innovation and implementation of digital interventions in healthcare, ongoing evaluations and improvements are vital to ensure values and needs of stakeholders are met, irrespectively of method or framework used.

Future research should evaluate the uptake and impact of digital behavior change interventions for weight loss maintenance on both individual and system levels, including new healthcare delivery models, with exploration of a range of outcomes including improvement of health, cost-effectiveness and scalability (21).^1^ Such explorations and continuous monitoring through iterative data collections (63), have the potential to improve knowledge and decision support related to how digital behavior change interventions can be optimized, integrated and implemented as effective and sustainable parts of healthcare practice. As measuring impact of innovations on health outcomes can be challenging due to time lags between innovation and uptake in health systems (26), new methods for early assessment of innovations might be called for.

Future research should also examine challenges, opportunities and impacts of technology transfer processes of publicly funded research-based innovation, as well as innovative public-private collaborations to promote health and health behavior change of people living with chronic conditions such as obesity. Exploration of adoption and implementation of digital health interventions through public-private partnerships could contribute with valuable knowledge about various aspects of service and system innovation to improve health outcomes.

## Conclusions

This manuscript illustrates how *service design* and *technology transfer* of research-based innovation can be used to facilitate stakeholder involvement, service innovation and implementation of a digital behavior change intervention for weight loss maintenance in healthcare. While *service design* can be useful to explore how a new digital intervention can be integrated and implemented into the health system and services, *technology transfer* processes can contribute to accelerating implementation of research-based innovations into practice. Both approaches rely on interdisciplinary stakeholder collaboration to optimize the fit between needs, technology and the intended context, and can be executed simultaneously to facilitate implementation and scale-up of digital health interventions.

## Data Availability

Data supporting the conclusions of this article are included in the manuscript/Supplementary material. For further inquiries, please contact the corresponding author.

## Footnotes

^1^Asbjørnsen RA, Børøsund E, Hjelmesæth J, Smedsrød ML, Ollivier M, Wentzel J, et al. Digital behavior change intervention for weight loss maintenance in adults with obesity: a feasibility pilot study of eCHANGE. Forthcoming.
