# Supplementary figures and images for: Implementation of a digital behavior change intervention (eCHANGE) for weight loss maintenance support: a service design and technology transfer approach
Source: Front Digit Health. 2024 Jul 2;6:1394599. doi: 10.3389/fdgth.2024.1394599 (PMC11249862; doi:10.3389/fdgth.2024.1394599)

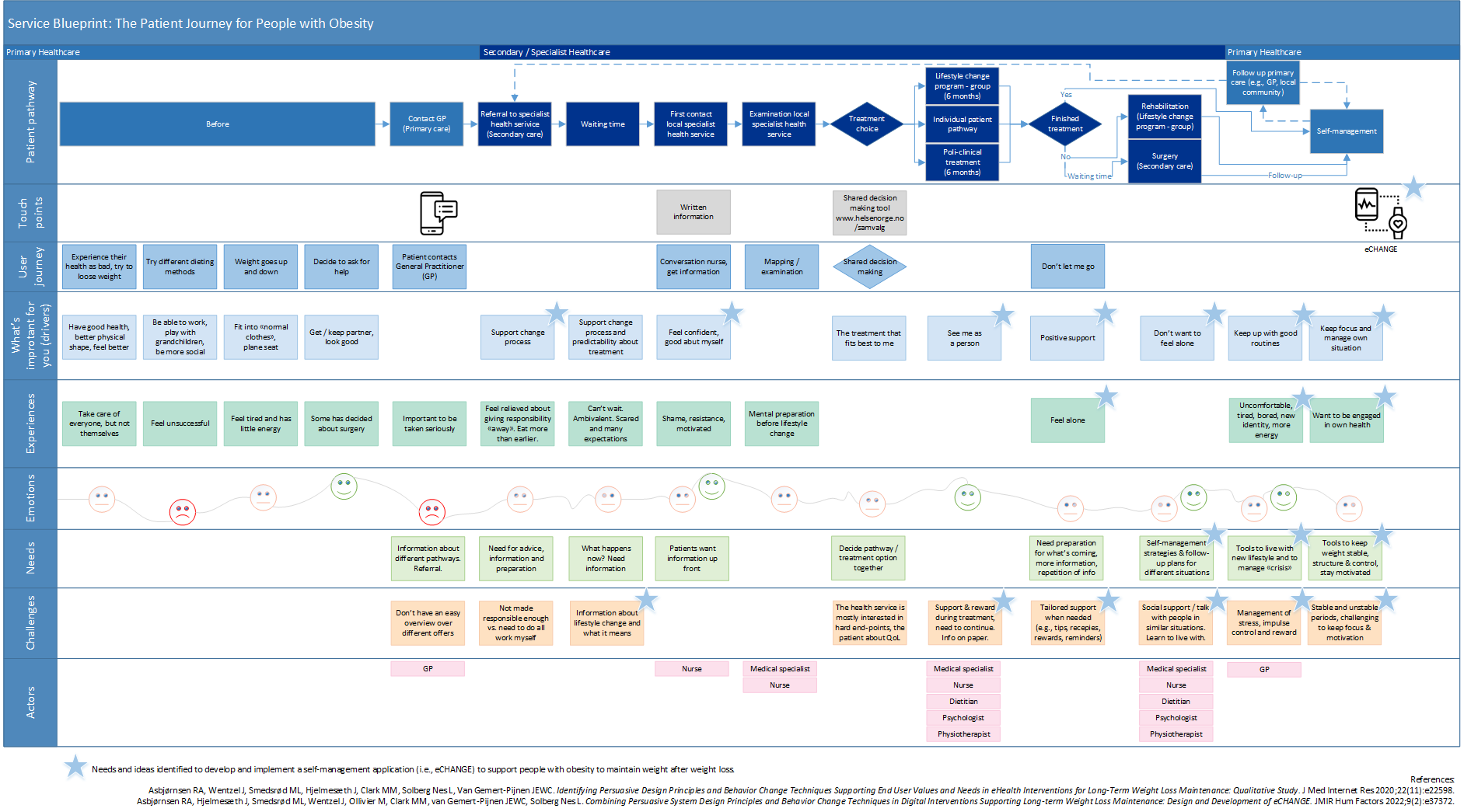

Supplement: Supplementary file 1 [file Image1.png]

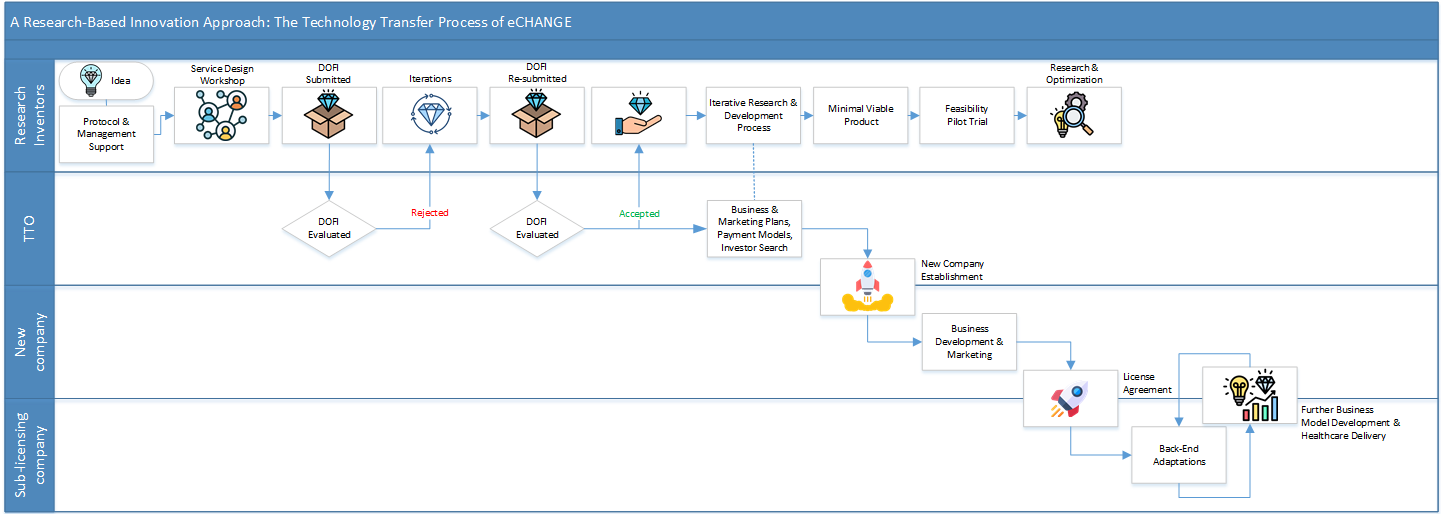

Supplement: Supplementary file 2 [file Image2.png]
